# A Framework for the Virtual Medical Interview Process: Considerations for the Applicant and the Interviewer

**DOI:** 10.31486/toj.21.0074

**Published:** 2022

**Authors:** Christina McCain, Brekel Kemp, Margaret Bishop Baier, Arnold H. Zea, Carl Sabottke, Emma R. Schachner, Claude Pirtle, Angela McLean, Robert Maupin, Pierre Detiege, Bradley Spieler

**Affiliations:** ^1^ School of Medicine, Louisiana State University Health Sciences Center, New Orleans, LA; ^2^ Department of Psychiatry, Louisiana State University Health Sciences Center, New Orleans, LA; ^3^ Department of Microbiology, Immunology, and Parasitology, Louisiana State University Health Sciences Center, New Orleans, LA; ^4^ Department of Medical Imaging, University of Arizona, College of Medicine, Tucson, AZ; ^5^ Department of Cell Biology and Anatomy, Louisiana State University Health Sciences Center, New Orleans, LA; ^6^ Department of Internal Medicine and Telehealth, West Tennessee Healthcare, Jackson, TN; ^7^ Office of Admissions, School of Medicine, and Department of Medicine, Louisiana State University Health Sciences Center, New Orleans, LA; ^8^ Department of Obstetrics and Gynecology, Louisiana State University Health Sciences Center, New Orleans, LA; ^9^ Department of Emergency Medicine, Louisiana State University Health Sciences Center, New Orleans, LA; ^10^ Department of Radiology, University Medical Center, Louisiana State University Health Sciences Center, New Orleans, LA

**Keywords:** *Academic medical centers*, *COVID-19*, *internship and residency*, *interviews*, *schools–medical*, *students–medical*, *videoconferencing*

## Abstract

**Background:** Videoconferencing platforms are being used for the purposes of interviewing in academic medicine because of the coronavirus disease 2019 pandemic. We present considerations applicable to interviewers and interviewees in the virtual space, with a focus on medical school and residency applicants.

**Methods:** We reviewed the literature regarding the virtual interview process for medical school and residency by searching PubMed using the following keywords and terms: “interview,” “academic medicine,” “medical school application,” “residency application,” “virtual interviews,” and “videoconferencing.” Our search identified 701 results, from which we selected 36 articles for review.

**Results:** The garnered information focuses on strategies for optimizing the virtual interview process from the standpoint of both the interviewer and the interviewee. We discuss the advantages and disadvantages of the virtual interview process and present recommendations.

**Conclusion:** While the future of the interview process for medical school and residency is uncertain, virtual interviewing is a common and growing practice that will continue to be at least part of the medical interview process for years to come. Interviewers and interviewees should prepare to adapt to the evolving changes in the process.

## INTRODUCTION

The coronavirus disease 2019 (COVID-19) pandemic has impacted all sectors of society, personal and professional alike. Social distancing efforts and travel restrictions^1,2^ have necessitated adaptions to traditional screening processes for medical school and residency training, most notably in the form of the virtual interview process. In March 2020, the Association of American Medical Colleges (AAMC) formally recommended that all interviews for potential students and trainees be conducted virtually.^3^ Similar recommendations for interviews related to graduate medical education were also made.^4^ Whether or not virtual interviewing will become a permanent component of the application process for medical training programs is unclear. Consequently, optimizing one's approach to the virtual space may be key to a successful application process moving forward.

We reviewed the literature regarding the virtual interview process for medical school and residency by searching PubMed using the following keywords and terms: “interview,” “academic medicine,” “medical school application,” “residency application,” “virtual interviews,” and “videoconferencing.” Our search identified 701 results, from which we selected 36 articles for review.

## ADVANTAGES OF VIRTUAL INTERVIEWS

This paradigm shift in the interview process has several advantages (Table 1). Paramount among them is the reduction in financial cost to the interviewee. Virtual interviewing eliminates the most expensive aspects of the interview process, namely travel and hotel expenses. Likewise, the elimination of travel reduces the time burden on applicants who generally feel obligated to attend as many interviews as possible but often encounter scheduling conflicts with their senior year curriculum and other interviews. Interviewers and interviewees alike are now partially relieved of juggling interview date preferences and delays in interview date confirmations because of the increased scheduling flexibility with virtual interviews.^5,6^ Additional benefits of virtual interviewing include active social media outreach by interviewing institutions and an introduction to virtual communication systems that are similar to the telehealth systems that future physicians will likely use.^7^

**Table 1.** Advantages and Disadvantages of the Virtual Interview

Advantages	Disadvantages
Reduced financial cost	Loss of casual interaction with current residents/students
Less time away from school and work	Decreased awareness of nonverbal cues/body language
Fewer conflicting interview times Fewer travel limitations	Natural conversation thwarted (delay/lag, difficult to interrupt, laughing competes with audio)
Greater diversity of interviewers because of fewer scheduling/travel difficulties	Technical difficulties creating a barrier to experience
	Loss of environment standardization (background, voice quality)
Preinterview outreach more available and accessible to applicants (social media, virtual information sessions)	Possible new sources of bias (applicant's perceived environment and comfort with technology)
Institutions forced to maintain their online presence and provide more online resources regarding their institution and city	Inability to gain detailed knowledge about the city, the program, and the institution
Introduction to the era of telehealth	

## DISADVANTAGES OF VIRTUAL INTERVIEWS

Despite the significant advantages of virtual interviews, the process has notable disadvantages, such as the inability to directly interact with members of an institution and to obtain a feel for the training environment.^8^ The training environment issues can be partially mitigated with multimedia. For example, well-designed virtual tours can show applicants the training facilities and the neighboring areas, including housing opportunities and testimonials from current students. Information ordinarily provided to applicants in person can be provided digitally.^9^ When a fellowship program at Newton-Wellesley Hospital in Massachusetts offered virtual tours, 85% of candidates found the virtual experience and digital materials gave them a satisfactory and sufficient understanding of the program; however, 17% still chose to visit the hospital in person after the interview.^10^

Other disadvantages are intrinsic to virtual interviewing. The virtual setting provides limited visualization of the other person, thereby reducing the transmission of nonverbal cues. Even with the best technology, the potential for poor synchronization of auditory and visual signals remains. These issues disrupt the natural flow of conversation. Studies suggest these factors may negatively influence both applicant impressions and interviewer ratings.^8^ The Healy and Bedair study underscored this drawback: 15% of resident candidates interviewing for an orthopedic fellowship by videoconference did not believe they had the opportunity to present themselves to their satisfaction.^10^

## BEFORE THE VIRTUAL INTERVIEW

To optimize their chances of success, medical applicants in past interview seasons built a rapport with program personnel before their formal interviews. Opportunities such as away or audition rotations and preinterview receptions were key to establishing facial recognition with residents and faculty before interview day. However, physical constraints imposed by the pandemic have resulted in programs forgoing these traditions out of necessity.^4^ Current and upcoming applicants must recognize this change and consider other possible avenues to connect with their desired programs remotely. This change may be advantageous to applicants who might not have had the means to travel to multiple destinations.

### Virtual Rotations

Various medical institutions have begun offering virtual rotations in place of the traditional away rotations. These rotations are important opportunities for students who are considering applying to these institutions. Although students will be unable to display the extent of their clinical skills in person, they can use these courses to virtually meet and communicate with residents and faculty in their desired program. Some virtual rotations are designed specifically for applicants, offering them a focused look at the program and an opportunity to express their interest.^11^ A small pilot study reported in fall 2020 offered virtual rotations to senior medical students interested in emergency medicine and received positive survey results; the students found the experience to be elucidative, enjoyable, and feasible.^12^ Virtual coursework has the potential to be as effective as in-person courses in educational impact.^13^

### Preinterview Events

The pandemic has similarly discouraged other typical preinterview events such as question and answer sessions (Q&As) and come-and-see days. Because of restrictions on travel and gathering, programs are largely opting for virtual equivalents to attract and maintain applicant interest. As with virtual away rotations, these events offer applicants a chance to directly communicate with program administrators and establish facial recognition before the official interview day. Such virtual meetings encourage flexible communication and give applicants the opportunity to engage with a number of programs.^14^ However, these events tend to be less structured than a rotation and last only one to several hours.

### Social Media

Program-sponsored events may be advertised directly through the program's website, outreach to applicant institutions and interest groups, and social media.^15^ The role of social media in the medical application process, especially in the preinterview stage, has evolved from supplementary to vital in the era of COVID-19.^16^ Meet and greets, Q&As, and information sessions are largely advertised and sometimes facilitated via platforms such as Twitter and Instagram. Social media presence is another compensation for the loss of away rotations.^16^

Regardless of whether an applicant plans to use a virtual platform as a means of direct engagement in the preinterview period, ensuring that all of one's personal social media accounts are updated and professionally acceptable is important. Personal information and statements published online will be factored into interviewee selection and into final application decisions. Program administrators often use social media as an informal background check and are likely to withhold interview slots from students with an online history of unprofessional language and behavior.^17^

### Program-Specific Resources

The shift to virtual interviews has prompted some institutions to update and expand their online resources.^18^ These resources include program homepages, frequently asked questions, virtual tours, and supplementary materials meant to compensate for the lack of in-person engagement on interview day. While these resources undoubtedly help with an applicant's decision-making, they also provide an opportunity to gauge applicants’ earnest interest in a program. Interviewees who review program resources and prepare relevant questions based on those materials make more positive impressions on their interviewers.^19^ Some programs provide tutorials and practice interview sessions for applicants.

### Practice Sessions

Regardless of whether formal practice sessions are offered by interviewing entities or a student's home institution, interview rehearsal has an undeniable benefit. Performance anxiety has the potential to negatively impact a virtual interview even more than an in-person interview.^20^ Virtual interviews can cause interviewee anxiety and frustration^21^ beyond what is normally encountered in traditional in-person interviews, potentially resulting in the interviewers’ perception that the applicant has limited social skills. In a study of medical students who agreed to video-record mock consultations, many cited a major source of their worries as “being thought to be inadequate in personality, or in basic communication skills.” However, this anxiety was shown to be exaggerated and preventable for future interview performance by participating in practice evaluations and receiving feedback.^22^

Rehearsal for a virtual interview should be conducted on a virtual recording platform, ideally the same platform used by the interviewing institution to best replicate the technical mechanics of an interview day. Familiarity and proficiency with the interviewing institution's videoconferencing software has been reported to reflect well on the interviewee's perceived organizational abilities.^23^

One method of interview rehearsal entails recording oneself answering questions within a set time interval, approximately 10 minutes or the time allotted for interview sessions at institutions of interest.^24^ Frequently asked interview questions may be supplied by mentors or obtained from online resources such as the AAMC.^25^ Once the recording is complete, both the interviewee and a trusted third party, such as a professor or mentor, should review it, with the third party providing feedback regarding the interviewee's physical presentation and responses. The interviewee should consider all feedback and repeat the rehearsal as many times as necessary to develop confidence for the interview period.

## TECHNICAL FACTORS

Although virtual interview rehearsal principally serves to hone an applicant's interview skills, it can also act as a test run of the applicant's technology setup. Interviewees are responsible for technical factors such as internet connection, audio settings, and consistent video quality. These factors all contribute to smooth communication with the interviewer and to the applicant's image.

### Internet Connection

Virtual interviews are generally held via conference platforms such as Cisco Webex or Zoom.^26^ Smooth use of these programs requires a seamless online network connection, so applicants should thoroughly test their connections in the environment where they plan to conduct their formal interviews.

The ideal setup for seamless streaming quality is a personal computer remotely connected to a secure Wi-Fi network and also manually connected to the internet via an ethernet cable. Although Wi-Fi alone may suffice, an ethernet connection is more reliable than Wi-Fi.^19^ The AAMC emphasizes speed and stability in their recommendations for interview day, noting that a wired ethernet connection can maximize the speed and strength of a connection.^27^ Devices such as cell phones are not recommended, as they depend on comparatively unreliable cellular networks and typically do not offer ethernet connectivity.^28^

However, no mode of internet connection is infallible. Applicants should have the technology help numbers supplied by their interviewing institution on hand throughout the interview day in case of technical difficulties they cannot personally resolve. Additionally, applicants should keep their cell phones nearby and in full charge in case the interview needs to be continued over the phone.

### Audio

Most computers are manufactured with an internal microphone. While these microphones are generally sufficient for casual online interaction, their lack of sound quality and consistency may be an obstacle to efficient use of interview time. Internal microphones are relatively small and therefore limited in their ability to capture voice. Many microphones compensate by amplifying harsh midrange frequencies to help the voice cut through other sounds, a mechanism that may be helpful with one speaker but may cause awkward cutoffs when two participants try to speak simultaneously. Consequently, the interviewer and interviewee may have to repeat themselves multiple times, wasting time during the interview or causing confusion.^29^ Applicants should therefore consider investing in a dedicated microphone for interview season. A high-quality microphone not only optimizes voice quality but also ensures that nonvoice interruptions are minimized. The elimination of ambient background noise cannot be undervalued, as unpredictable background noises have been reported to cause significant setbacks. For example, one fellowship program's interview day was derailed by the triggering of a fire alarm in the main speaker's building.^30^

## OTHER RESOURCES FOR INTERVIEW DAY

Having tools such as an external microphone and a dedicated quiet space at home may not be feasible for all applicants. Students commonly live in shared quarters where sound levels are out of their control, and adequate technology and a seamless internet connection may not be financially within reach. In such cases, applicants should consult their home institutions regarding the resources available to them during interview season. The AAMC recommends requesting reservations from the interviewee's medical school or local library for a private interview room/space that will provide a quiet setting and reliable internet connection.^27^ Some locations offer equipment lending services (ie, computer rentals, microphones) that can help to minimize the impact of economic disparities among applicants.^31^

## DURING THE INTERVIEW

### Personal Considerations

An important aspect of social interaction is how we perceive the appearance of others.^32^ In radiology programs and beyond, perceived attractiveness has been shown to have a similar correlation to success as nonphysical factors such as the United States Medical Licensing Examination Step 1 score.^33^ Interviewees should dress as formally as they would for an in-person interview; attire should never be the focus of the interview. While the face and torso are the main focal point during a virtual interview, interviewees may need to stand up while on camera, so they should wear a complete professional outfit. Applicants should consider testing their interview day outfit, as certain colors may blend with the background or may not be flattering on screen. A classic white shirt may wash out on screen. Patterns and bulky accessories can be distracting. Accessories can also create noise; even a dainty bracelet can make an intrusive sound when hit against a computer or desk.

**Figure 1.**
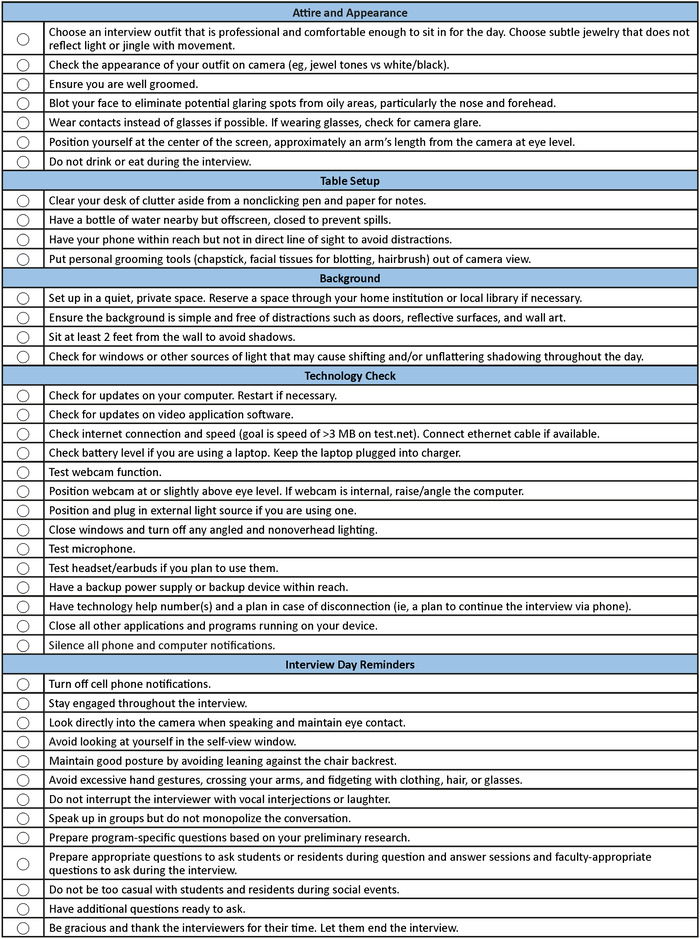
Interviewee checklist inspired by resources from the Association of American Medical Colleges.^27^

Traditional interview attire standards apply in the virtual setting, as well as the general tips from the AAMC (Figure 1).^27,34^ These general tips provide a flexible framework for applicants, but interviewers must be mindful of potential implicit bias,^35^ as applicants may present with appearances based on culturally rooted preferences or values that may be unfamiliar to the interviewers. Recognition of implicit bias based on an interviewee's appearance^36^ can aid in redirecting an interviewer's focus to the substance of the interview rather than the interviewee's expression of personal identity.

Lighting is important. Overhead lighting is generally sufficient.^19^ Bright lights directly behind the interviewee are likely to cast a shadow. For optimal lighting, ring lights and other lighting tools traditionally used in photography can be used. A ring light is a circular light that provides even illumination across the face when positioned appropriately. The light should be positioned so it is centered in front of the face, not above or below, to avoid unfavorable shadows. Ring lights or other external light devices should be fully charged or plugged into an outlet.

In addition to being mindful of physical appearance, applicants need to be aware of potentially distracting behaviors and avoid them. Anecdotal examples noted by interviewers include the following:

- Adjusting hair, tie, and glasses
- Reading notes from the screen or from paper
- Gum chewing, eating, or excessively drinking water
- Pen tapping/clicking
- Glare/reflection of eyeglasses
- Rocking/swiveling in a desk chair
- Staring elsewhere on the monitor rather than at the camera^23^

Applicants should also be conscious of unnecessary vocal interjections. Laughing aloud, in response to an interviewer's joke or comment, may be natural and appropriate in certain settings; however, in the virtual setting laughter can quickly become competing audio and may cut the interviewer off prematurely. Similarly, rather than using casual verbal interjections, such as “yeah” or “mm-hmm,” to demonstrate agreement, applicants should rely on nonverbal cues such as head nodding to avoid accidentally interrupting the interviewer. Similarly, excessive hand gestures should be avoided when speaking, as any lag time will cause the gestures to stutter on screen.

Interviewees should avoid asking questions or introducing new topics when 1 minute remains in a session. The 1-minute mark is a good time to wrap up the interview session to avoid an awkward conclusion and to ensure the interview is completed on time. Muting the microphone and closing the video session once the interview is completed may help prevent potential awkwardness after the close of the interview.^37^

Appearing engaged and attentive throughout the discussion is crucial, particularly in the virtual setting because an applicant can easily appear to be distracted, given the limited view provided on the screen. A strategy to mitigate this issue is to maintain direct eye contact. In interviews held by Mayo Clinic's internal medicine residency program in Arizona, successful interviewees had their cameras positioned directly at eye level, and interviewees appeared to consistently maintain eye contact.^19^ This eye contact was perceived as active engagement throughout the interview. Lack of eye contact in conversation has been associated with a negative impact upon information recall,^38^ which could be disadvantageous to applicants interviewing in the virtual space where eye contact can be hampered by technical factors^39^ and some neurophenotypes.^40^

**Figure 2.**
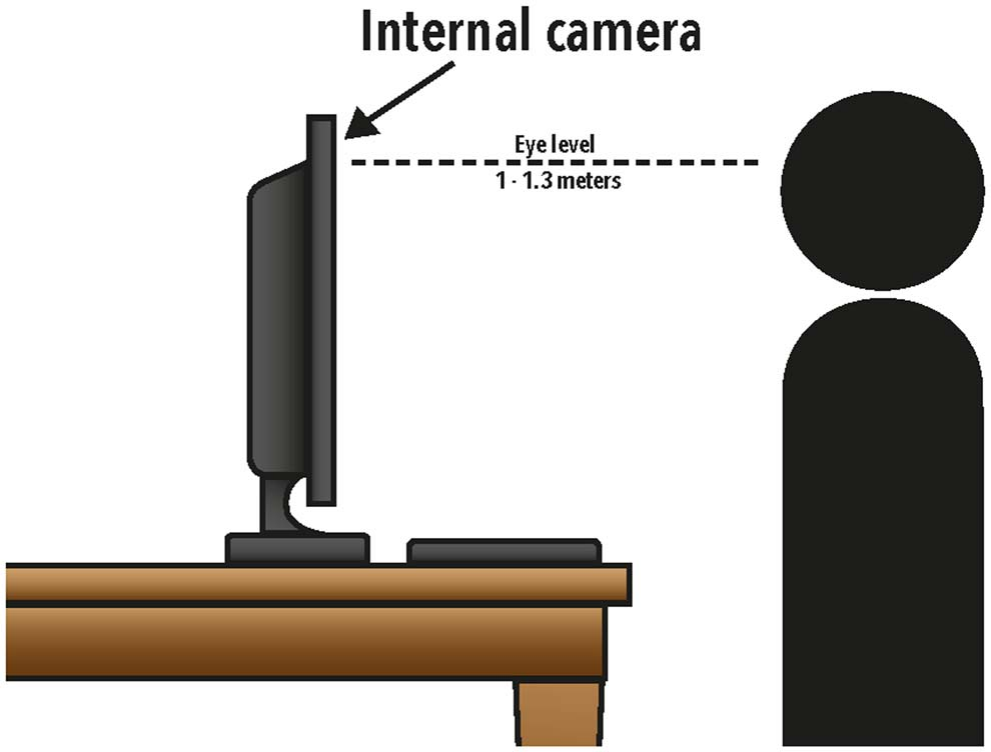
Suggested position of an applicant relative to the computer for a virtual interview.

The following tips can help with perceived direct eye contact:

- Interviewees should sit approximately 1.3 meters from the webcam, with the webcam directly at eye level.
- Webcams should be positioned to crop the field of view of the head above the hairline with the face centered on the screen (Figure 2).
- Laptops should be placed on an inclined surface.^41^

### Physical Environment

The interview environment should be quiet and free of potential distractions. A neutral, blank background will prevent distractions and reflect professionalism. Clutter, doors, moving objects, and reflective surfaces should not be in the background. An outlet should be nearby in case a device needs to be charged. Windows and doors should be closed to minimize noise. Discussing the upcoming interview with neighbors and roommates/family can help ensure minimal excess noise.^42^ If other individuals or pets may be a potential contributor to environmental noise, interviewees should generate a plan to keep them occupied during the interview. If possible, interviewees should avoid spaces where they lack control over background noises or may be interrupted.

**Figure 3.**
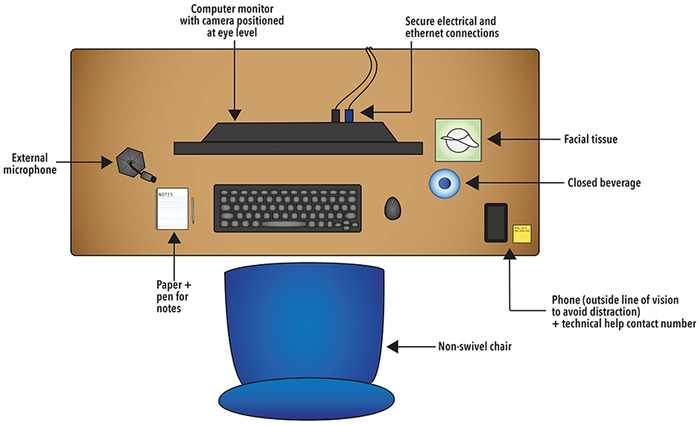
Suggested desk setup for a virtual interview.

Desks should be clear of items other than minimal notes, blank paper, and a pen for taking notes. Anyone prone to fidgeting should not use a retractable pen. Water should be out of direct view of the camera and sipped only between interviews or during breaks. Some drinkware may loudly clink when set down, and ice can also create a distracting sound. Figure 3 shows a suggested setup for the virtual interview.

In addition to environmental factors, applicants must ensure their technology setup is maximized for virtual interviewing by conducting a technology check. The computer should be fully charged with a power source nearby. Unnecessary programs or webpages should be closed. Updates for the computer and the video software application (eg, Zoom, Cisco WebEx, Microsoft Teams, Skype) should be installed in advance of the interview and the computer restarted after the updates. Sound notifications on the computer and phone should be silenced, as notification sounds can be transferred through the microphone and be distracting. However, the phone should be turned on and nearby in case a technology-related issue occurs, and the applicant needs to connect with an emergency contact or complete the remainder of the interview by phone. A checklist can be useful to prepare for potential technology difficulties (Figure 1). Applicants should follow up with interviewers immediately if technical difficulties occurred. If technical difficulties severely obstructed interview performance, the applicant may contact the interviewer or ask if the interviewing institution has a protocol for repeat interviews.

## CAUTIONARY CONSIDERATIONS

### Technical Bias

Virtual interview quality is a growing concern. In a 2018 study, candidates with high-quality videos were rated higher on their interviews than candidates with perceived lower quality video, suggesting that hiring decisions may be influenced by the interviewee's technological device or internet connection.^43^ This potential bias could be a concern for applicants with limited access to high-speed internet connectivity, such as in rural and underserved areas. Despite virtual interview benefits such as reduced cost, elimination of travel, and greater feasibility in managing the interview timeline, a 2020 study indicates that medical students and residents still favor the in-person format.^44^ A meta-analysis by Blacksmith et al noted that interview scores can be influenced by technical aspects in the virtual interview space.^45^ The investigators concluded that electronic conferencing platforms can alter both interview conduct and perceptions. A key factor in this process may be the limitation on *impression management*, the ability of interviewees to affect interviewers’ impressions of them through nonverbal conduct, speech patterns, and responses to visual cues.^46^ Technical factors such as internet connectivity and variable camera-related fields of view may be contributory.^21,47^ This point is important, as effective impression management tactics have been associated with higher interview scores.^48^

### A Culture of Inclusivity in the Virtual Space

As the generation of applicants being interviewed virtually evolves, setting a precedent and leveling the interview playing field are imperative in fostering a culture of inclusivity among the applicant pools. At the highest level of government in the United States, policies that endorse a culture of inclusivity help optimize engagement and increase the ability to select from the widest possible range of talent.^49^ The declaration of one's preferred pronoun (Table 2)^50^ is a way in which a culture of inclusivity can be championed in the virtual interview space. The designation of a preferred pronoun can serve as a marker that the virtual interview space is one of open-mindedness and acceptance. This simple gesture, which can be exercised by inserting one's preferred pronoun next to one's name on the video conferencing platform used for the interview, has been associated with interviewees’ positive perception of support and decreased stress during the interview.^51^ Such initiatives could be integral in catalyzing an ethos of diversity and inclusion, not only for the interviewers’ program but also for the institution.^52^

**Table 2.** Gender Pronouns

Subjective	Objective	Possessive	Reflexive	Gender Neutral?	Example
He	Him	His	Himself	No	He bought his books himself.
She	Her	Hers	Herself	No	She bought her books herself.
They	Them	Theirs	Themselves	Yes	They bought their books themselves.
Ze	Hir/Zir	Hirs/Zirs	Hirself/Zirself	Yes	Ze bought hir/zir books hirself/zirself.

Note: Adapted from resources by the Stonewall Center at the University of Massachusetts Amherst.^50^

### Privacy and Security

The transition to a virtual interview format raises potential issues related to security and privacy. One issue is the control of the data generated by a virtual interview. If teleconferencing software such as Zoom is used for a synchronous interview, the option to record the interview is embedded within the software suite. Interview recording may seem advantageous for the institution if members of the interviewing committee have markedly different impressions or scores for an applicant. Members of the committee can review the interview session to help better assess the candidate. However, current guidance from the AAMC regarding maintenance of student records is that American Medical College Application Service applications be maintained for only 5 years after an individual's graduation or separation from the institution.^53^ If an institution chooses to record interviews, appropriate safeguards need to be in place to maintain confidentiality and prevent unauthorized access of the video record.

An institution may choose not to record interview sessions for several reasons. Interviewers may not want to risk interview questions being leaked to future candidates. Alternatively, if interview questions vary considerably between candidates in a nonstandardized way, the institution may not wish for candidates to compare questions or potentially perceive bias.

Even if an institution does not want to record interviews, the virtual format presents a unique obstacle. Most teleconferencing software has the option to disable meeting recording, but candidates can still record the interview on their personal computers using screen capture software such as ActivePresenter (Atomi Systems Inc) or OBS Studio (OBS Project).

For residency interviews, recording may change how an institution approaches interview questions that the National Resident Matching Program (NRMP) considers illegal or coercive, such as those related to age, gender, race, religion, sexual orientation, and family status.^54^ If an institution's interviewing committee does not know if a virtual interview is being recorded, committee members may hesitate to discuss topics that may potentially overlap with the NRMP's list of illegal or coercive questions. Ninety percent of medical students reported being asked at least one potentially discriminatory question during a residency interview.^55^

Another consideration is the intrusion of uninvited participants to the videoconference platform, sometimes referred to as “Zoom-bombers,” who may potentially listen in or otherwise disrupt the interview.^56^ Such interruptions could result in personal data being made publicly visible. Using the waiting room function of the videoconferencing software, ensuring that the meeting link is not public, and using a complex password can decrease opportunities for this occurrence.^26^

## CONCLUSION

The transition of medical interviewing from in-person to virtual was an abrupt departure from the traditional process applicants have been trained to navigate. We have provided a guide for applicants and interviewers for optimizing the virtual process. We have delineated the advantages and disadvantages of the virtual interview and provided specific strategies to mitigate some of the disadvantages. Although much is still uncertain about the future, virtual interviewing is now a common practice that will remain at least a fractional component of the medical interview process for years to come. Individuals on both sides of the screen must be ready to adapt to these changes.

## Notes

*This article meets the Accreditation Council for Graduate Medical Education and the American Board of Medical Specialties Maintenance of Certification competencies for Interpersonal and Communication Skills and Practice-Based Learning and Improvement.*
